# Cefepime-Induced Neurotoxicity

**DOI:** 10.7759/cureus.17831

**Published:** 2021-09-08

**Authors:** Tanjot Saini, Monica N Gaines, Aalam Sohal, Lin Li

**Affiliations:** 1 Internal Medicine, University of California, San Francisco, Fresno, USA

**Keywords:** cefepime induced neurotoxicity, kidney failure, kidney transplant, non-convulsive status epilepticus, pseudomonas infections, postoperative delirium, hemodialysis, cefepime, cefepime-induced neurotoxicity, cefepime-induced seizures

## Abstract

Cefepime is a common antibiotic used to treat various infections such as pneumonia, skin infections, and intra-abdominal infections due to its broad gram-positive and gram-negative spectrum. However, patients with acute kidney injury, end-stage renal disease, and renal transplantation are disproportionately at higher risk of developing complications from administration of cefepime, secondary to its predominant renal excretion. Current guidelines prescribe cefepime renal-dosing, dependent on the glomerular filtration rate, to prevent toxicity. This study presents a rare case where an acutely hospitalized patient undergoing chronic renal transplant rejection was administered renal-dose cefepime. Despite renal dosing, the patient developed neurotoxicity that manifested as delirium, inability to tolerate oral intake, and non-convulsive status epilepticus. Solely adjusting for renal dysfunction may be inadequate to prevent the accumulation of cefepime metabolites, which may present in an atypical manner in the patient. Such possibilities emphasize the need for continued evaluation of a patient’s mentation in case of cefepime administration. Cefepime-induced neurotoxicity incidences need to be evaluated and researched thoroughly.

## Introduction

Cefepime is a fourth-generation cephalosporin, which is excreted primarily by the kidneys. Cefepime-induced neurotoxicity is a well-documented adverse effect in patients with renal failure. Symptoms are correlated with decreased cefepime clearance due to reduced glomerular filtration rate (GFR) as well as increased central nervous system (CNS) penetration due to blood-brain barrier dysfunction [1]. The symptoms include depressed consciousness, encephalopathy, aphasia, myoclonus, and seizures [2]. While the mechanism of action behind this phenomenon is not well understood, it is thought to be related to concentration-dependent gamma-aminobutyric acid (GABA) antagonism. Most of the neurotoxicity case reports are associated with inappropriate dosing of cefepime [1]. However, a minority of cases reported (<25%) occur despite appropriate dosing of the medication. Typically, treatment involves discontinuation of the precipitating drug [3]. Here we present a unique case of a 64-year-old woman with renal failure who became altered on day six in the hospital due to non-convulsive status epilepticus while receiving renally dosed cefepime. 

## Case presentation

A 64-year‐old caucasian woman with a past medical history of scleroderma with pulmonary fibrosis, renal transplant 18 years ago, chronic pericardial effusion, and hypertension presented to the hospital with two days of right-sided neck pain and stiffness associated with numbness and tingling of her hands. Preliminary work-up was remarkable for an acute kidney injury with severe electrolyte derangements including hypocalcemia, hypomagnesemia, and hyponatremia. After two days of aggressive electrolyte repletion, the patient’s symptoms resolved, but kidney function continued to slowly deteriorate. The regional transplant center was contacted in coordination with the hospital nephrology team who deemed this as an acute chronic renal transplant rejection. 

The patient began to develop progressively worsening urinary retention requiring repeated straight catheters and eventually a foley. During this time she began to develop diffuse abdominal pain prompting an infectious work-up and resulting in two blood cultures and urine culture being positive for *Pseudomonas aeroginosa*. A ten-day course of cefepime was started for the concurrent infection with consideration of hemodialysis being initiated in the setting of continued worsening renal function. The infectious disease team was consulted for further recommendations given the patient's immunocompromised state and de-escalation of antibiotics with cultures being pan-sensitive. 

**Figure 1:**
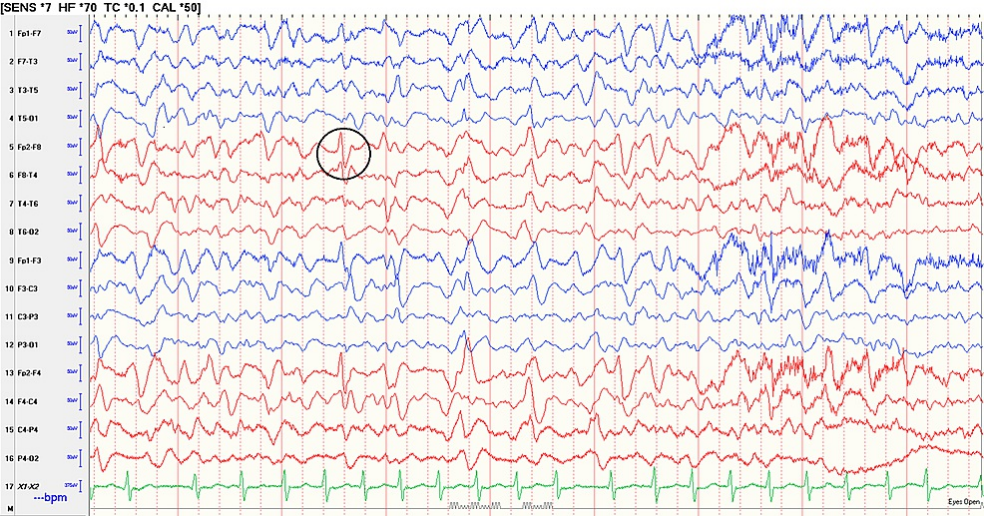
Electroencephalography (EEG) The black encircled area shows triphasic waves rather than frontal sharp waves that are most likely consistent with sub-clinical status epilepticus

On day six of cefepime administration, the patient developed acute delirium after undergoing placement of a tunneled dialysis catheter. Initially, the delirium was attributed to side effects of sedation, but the delirium began to worsen. She was interestingly always able to answer the standard orientation questions and partake in a linear conversation but developed fluctuating mental status. This was emphasized by her husband who endorsed unusual conversations with his wife and was further evident by inappropriate effects including hysterical laughter. Infectious work-up and computed tomography of the head were unrevealing. In light of the worsening mental status and progressively decreasing oral intake, an electroencephalogram (EEG) was ordered to further assess the altered mental status (Figure 1). 

EEG was remarkable for subclinical status epilepticus. The patient was loaded with levetiracetam and put on maintenance dosing for the remainder of the admission. Cefepime was discontinued and meropenem was started under infectious disease recommendations due to reported rare side effects of altered mental status from cefepime. 

Over the course of the next three days, the patient's mental status, oral intake, and hemodynamic stability improved remarkably. At her outpatient primary care physician appointment two weeks later she was reported to be doing well and had no associated complaints or confusion.

## Discussion

Cefepime, a common fourth-generation cephalosporin, has been reported in the literature due to its ability to cause neurotoxicity particularly in patients with renal failure. While the mechanism of cefepime-induced neurotoxicity (CIN) is still being researched, it has been hypothesized in the literature to be related to a decrease in GABA release from nerve terminals through a mechanism that is not fully understood, resulting in hyperexcitation of the neurons and depolarization of the postsynaptic membrane [1]. This manifests clinically as seizures, myoclonus, and encephalopathy [4].

It was first reported in the literature in 1999 in a patient with end-stage renal disease who was found to have high cefepime levels and subsequently developed altered mental status, myoclonus, and generalized tonic-clonic seizures [2]. Cefepime-induced neurotoxicity (CIN) occurs primarily in patients with renal dysfunction as the antibiotic is primarily renally excreted. For this reason, the U.S. Food and Drug Administration has recommended that the drug be renally-dosed [5]. Other risk factors for neurotoxicity include inappropriate dosing of the drug, previous brain injury (due to CNS penetration), older age, and disruption of the blood-brain barrier secondary to sepsis, uremia, or CNS infection [3].

Symptoms of CIN, including encephalopathy, seizures, and EEG changes, generally begin to appear approximately four days after initiation of the antibiotic. Encephalopathy generally manifests as tremor, aphasia, myoclonus, drowsiness, stupor, coma, confusion, delirium, and agitation. In a minority of cases (approximately 13%), a seizure was the only isolated symptom [3,5]. 

Diagnosis of CIN is made based on neurological symptoms starting days after cefepime initiation, related EEG findings consistent with generalized periodic discharges with a triphasic wave pattern, and resolution of symptoms and EEG abnormalities following discontinuation of the medication. Furthermore, CIN is a diagnosis of exclusion, and other causes of toxic and metabolic encephalopathy must be ruled out prior to making the diagnosis [5]. 

Treatment of CIN involves discontinuation of the drug. In a minority of cases with severe presentations, hemodialysis has been initiated for rapid removal of the drug [3]. Antiepileptic drugs are not indicated unless the patient is presenting with convulsive seizures or nonconvulsive status epilepticus [5]. Resolution of symptoms and EEG abnormalities should be expected approximately after one to three days following discontinuation of cefepime [3].

Our case presents a patient with acute renal failure requiring hemodialysis treated with renally-dosed cefepime for coverage of her *Pseudomonas* bacteremia secondary to a urinary tract infection. CIN developed approximately six days following the initiation of cefepime, manifested by encephalopathy and EEG abnormalities consistent with subclinical status epilepticus. Other organic causes including metabolic etiologies and stroke were ruled out. Three days after discontinuation of cefepime, the patient demonstrated an increase in oral intake and returned to her baseline mental status.

This case adds to the compilation of literature surrounding CIN due to its unique presentation of encephalopathy and EEG abnormalities in a patient with acute renal failure who received appropriate renally-dosed cefepime. Previous literature surrounding CIN focused on inappropriate renal dosing of cefepime as the major risk factor for neurotoxicity. In our patient, Cefepime was reduced by 50-75% the recommended dose in accordance with her decreased creatinine clearance. Nonetheless, the patient developed signs and symptoms of CIN within the expected time frame, which resolved appropriately with discontinuation of the antibiotic. We theorize that CIN may have developed in our patient despite appropriate dosing due to both her older age and disruption of the blood-brain barrier due to uremia and sepsis.

While the majority of cases regarding CIN have focused on inappropriate dosing of cefepime as the major risk factor, this case demonstrates that cefepime-induced neurotoxicity can occur in instances where cefepime is appropriately renally-dosed. It is important to understand this risk when initiating cefepime in an individual with renal failure, and have a low threshold for discontinuation of the medication if the patient begins to demonstrate signs or symptoms of CIN. Future research surrounding the incidence and severity of renally dosed cefepime precipitating CIN is warranted given the increasing use of the antibiotic for the treatment of sepsis.

## Conclusions

Recognizing cefepime induced neurotoxicity can be a challenging due to a multitude of factors that more commonly are associated with encephalopathy and seizures. However, in the absence of other discernable sources, cefepime-induced toxicity should be considered as a diagnosis of exclusion. Dose adjustment can reduce the incidence of toxicity, but without close monitoring even therapeutic levels can lead to neurotoxicity. While this is one independent case at our institution, further research is needed to identify risk factors other than poor renal function to help recognize the true incidence and assist clinicians in their management.

## References

[1] Sugimoto M, Uchida I, Mashimo T (2003). Evidence for the involvement of GABA(A) receptor blockade in convulsions induced by cephalosporins. Neuropharmacology.

[2] Wong KM, Chan WK, Chan YH, Li CS (1999). Cefepime-related neurotoxicity in a haemodialysis patient. Nephrol Dial Transplant.

[3] Lee Lee, S S (2019). Cefepime-induced neurotoxicity. J Neurocrit Care.

[4] Payne LE, Gagnon DJ, Riker RR, Seder DB, Glisic EK, Morris JG, Fraser GL (2017). Cefepime-induced neurotoxicity: a systematic review. Crit Care.

[5] Appa AA, Jain R, Rakita RM, Hakimian S, Pottinger PS (2017). Characterizing cefepime neurotoxicity: a systematic review. Open Forum Infect Dis.

